# An Anomalous Left Main Coronary Artery Arising From the Right Sinus of Valsalva With Anginal Chest Pain

**DOI:** 10.7759/cureus.41773

**Published:** 2023-07-12

**Authors:** Jason Corcoran, Mahmoud Barbarawi

**Affiliations:** 1 Research, Alabama College of Osteopathic Medicine, Dothan, USA; 2 Cardiology, University of Connecticut School of Medicine, Farmington, USA

**Keywords:** right sinus of valsalva, angina, sudden cardiac death, coronary computed tomography angiography, anomalous coronary artery

## Abstract

An anomalous coronary artery (ACA) is a congenital malformation or variation where one or both coronary arteries have an abnormal origin. This condition has been associated with a high risk of adverse cardiac events, including sudden cardiac death. Our patient initially presented nine years before the diagnosis of the ACA with anginal chest pain on exertion. The patient had positive nuclear stress with both ST depressions and ST elevations, as well as transient ischemic dilatation of 1.36. A coronary artery angiogram revealed an anomalous left coronary artery originating from the right coronary sinus. The distal anatomy was determined with coronary computed tomography angiography (CCTA), which showed an interarterial course. The patient underwent coronary artery bypass surgery following CCTA.

## Introduction

An anomalous coronary artery (ACA) is a congenital malformation or variation where one or both coronary arteries have an abnormal origin. The prevalence of an ACA has been reported anywhere from 0.3% to 2% in the general public, which has increased with the widespread use of invasive and noninvasive imaging for ischemic heart disease [1-4]. Because most ACAs are asymptomatic, they are usually incidental findings during the workup of another cause of ischemic heart disease. Invasive coronary artery angiogram (ICA) has been considered the most important tool in identifying and classifying ACAs; however, coronary computed tomography angiography (CCTA) has emerged recently as a diagnostic tool due to its less invasive and three-dimensional nature [5]. Anomalies of origination and course of the coronary arteries can be classified into one of the following five categories: absent left main trunk, anomalous location of coronary ostium within the aortic root or near proper aortic sinus of Valsalva, anomalous origin of coronary ostium outside normal coronary aortic sinus, anomalous location of coronary ostium at improper sinus, and a single coronary artery [6]. One of the most clinically significant subvariants of the ACA is a left coronary artery originating from the right coronary sinus with an interarterial course [7,8]. In this variation, the left main coronary artery originates from the right coronary sinus and travels between the ascending aorta and the pulmonary artery before bifurcating into the left anterior descending and left circumflex arteries [3]. This variation has been associated with a higher risk of cardiac angina, ventricular arrhythmia, and sudden cardiac death in young adults [9-11]. In this case report, we present a patient with a history of long-standing angina who was found to have an interarterial anomalous left main coronary artery originating from the right coronary sinus.

## Case presentation

The patient is a 47-year-old female who presented to her cardiologist with a complaint of intermittent chest pain for the past nine years. The patient has a history of rheumatic fever, hyperlipidemia, and bilateral carpal tunnel syndrome and has undergone a cholecystectomy.

Nine years before her last presentation, the patient presented to her cardiologist with a complaint of chest discomfort that she described as an intense sensation over her throat, jaw, chest, and arms as well as dizziness and feelings of both hot and cold. At that time, an EKG was performed, revealing no abnormalities, and the patient's vital signs were within normal limits. She underwent a treadmill stress test, which did not show any obvious signs of ischemia. Additionally, a loop monitor was used to track her heart rhythm for one month, and no significant arrhythmias were detected. Three years after her initial presentation, the patient followed up with her cardiologist for preoperative evaluation before undergoing cholecystectomy. Once again, her EKG showed no abnormalities, and her vital signs were within normal limits. A repeat treadmill stress test was conducted, which revealed no abnormalities. Furthermore, an echocardiogram was performed, yielding unremarkable results.

The patient’s anginal chest pain continued to worsen, and she was experiencing it with very minimal activity. Then, at her last presentation, a nuclear stress test was performed. The patient exercised for seven minutes, completing stage 2, reaching a peak heart rate of 139 beats per minute (bpm) (81% predicted maximum heart rate), and achieved an effort of 9.6 metabolic equivalents (METs). The patient reported no symptoms; however, the study was terminated due to significant EKG findings. The EKG findings demonstrated a heart rate of 102 bpm with 3-4 mm ST segment depressions in the limb leads II, III, and aVF as well as chest leads V4-V6. ST-segment elevations were also noted in aVR and aVL. During stress testing, the patient’s blood pressure also dropped with exercise. Stress and rest nuclear images revealed a large area of anterior, anteroseptal, and apical walls suspicious of moderate ischemia. Finally, a transient ischemic dilatation (TID) during stress was noted at 1.36.

**Figure 1:**
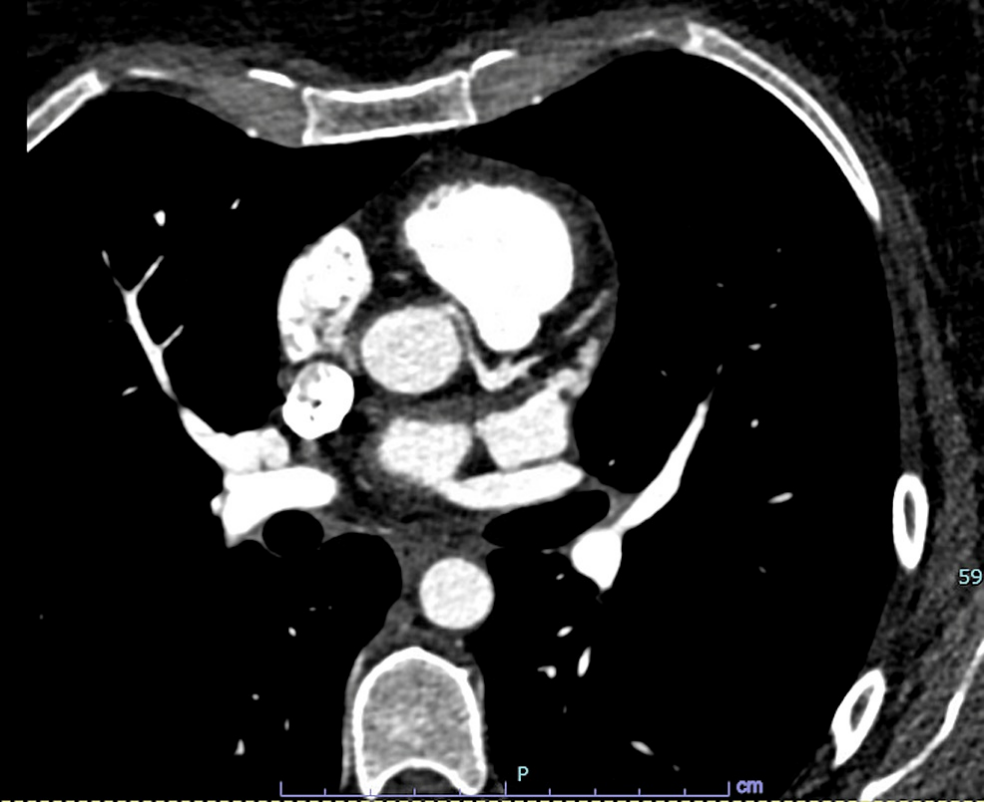
CT Angiogram Demonstrating the Interarterial Course of the Left Main Coronary Artery

The patient was immediately referred for left heart catheterization. Coronary catheterization of the right coronary artery was performed, which showed a large-caliber dominant vessel originating from the right coronary cusp. No significant lesions were noted in the right coronary artery or its territory. Multiple attempts to engage the left main coronary artery were made without success. A nonselective shot was taken in which significant stenosis was appreciated in the ostial left main coronary artery. Subsequently, an aortogram was performed in which >70% stenosis was appreciated in the ostial left main artery. The aortogram provided evidence that the left main coronary artery originated from the right coronary cusp. To confirm the diagnosis and determine the distal path of the left main coronary artery, a CT angiogram was performed. The CT angiogram demonstrated that the left main coronary artery traversed between the ascending aorta and the pulmonary trunk (see Figure 1). Based on the findings from the CT angiogram, the patient was referred for coronary artery bypass surgery.

## Discussion

Anomalous left coronary arteries originating from the right coronary sinus are associated with several complications, including sudden cardiac death [8,12,13]. Coronary artery anomaly has been reported as the second most common cause of sudden cardiac death in younger adults, with hypertrophic cardiomyopathy as the most common cause [14]. The current recommendation for young athletes presenting with anginal chest pain is the routine physical and echocardiographic examination. Some research has advocated the use of cardiac CT imaging as the next diagnostic approach if a coronary artery anomaly is suspected [15,16].

While the prevalence of coronary artery anomaly is reported from 0.3% to 2% in the general public, anomalous origin of the left main coronary artery originating from the right sinus of Valsalva is rare, with a reported prevalence of 0.017-0.03% [1,14]. The left main coronary artery originating from the right sinus of Valsalva is considered one of the most clinically significant anomalies due to its association with sudden cardiac death. It was postulated that this is due to the aorta pinching the left main coronary artery against the pulmonary artery, causing ischemia. More recently, it has been postulated that ischemia may also be caused by the acute angle causing non-atherosclerotic ostial stenosis [9]. Expansion of the aorta during exercise can compress the lumen of the left main coronary artery and worsen the stenosis, compromising blood flow. This results in myocardial ischemia and can lead to arrhythmia and sudden cardiac death. Approximately 30% of athletes who die of sudden cardiac death have been reported to have symptoms such as chest pain, shortness of breath, palpitations, presyncope, and syncope leading up to sudden cardiac death [14]. Not all variations of coronary artery anomalies present with symptoms of anginal chest pain; however, they are reported much more frequently in anomalies that involve an interarterial course of the left main coronary artery than in patients with benign anatomical anomalies. In treating patients with these symptoms, the possibility of coronary artery anomaly should be considered, and proper examination should be performed, including coronary artery angiography or CT angiogram.

Most of the published reports concerning left main interarterial anomaly presented in medical emergencies with cardiovascular compromise [17,18]. To the best of our knowledge, no reported case is similar to the current patient regarding clinical presentation of long-standing anginal chest pain on exertion due to the left main coronary artery between the aortic root and pulmonary artery trunk detected via nuclear stress testing.

Recognition of coronary arteries and definition of the anatomy is imperative in properly treating ACAs to prevent further symptoms and possible sudden cardiac death. Coronary artery angiography is currently the standard diagnostic imaging for coronary artery anatomy, with coronary CT as a complement. In our patient, coronary CT was performed to safely define the course of the left main coronary artery after it was determined to originate from the right sinus of Valsalva. Coronary artery bypass grafting was performed allowing bypassing of the interarterial segment that is believed to cause the stenosis responsible for the patient's symptoms.

## Conclusions

This patient presented with a long-standing history of angina on exertion with several normal stress tests prior to the last abnormal nuclear cardiac stress test. A coronary artery angiogram revealed a left coronary artery originating from the right coronary cusp with an interarterial course. Such cases should be documented in detail as they may influence the workup of anginal chest pain and could possibly prevent complications such as sudden cardiac death.
